# Distinct viral clades of SARS‐CoV‐2: Implications for modeling of viral spread

**DOI:** 10.1002/jmv.25902

**Published:** 2020-06-24

**Authors:** Adam Brufsky

**Affiliations:** ^1^ Department of Medicine, Division of Hematology‐Oncology, UPMC Hillman Cancer Center, Magee Women's Hospital University of Pittsburgh School of Medicine Pittsburgh Pennsylvania

A current model of the coronavirus disease 2019 (COVID‐19) epidemic^1^ appears to have differing degrees of projected total deaths in various areas of the United States despite the close timing of social distancing measures. For example, California with a population of approximately 40 000 000, began social distancing measures on 19 March 2020, yet is projected to plateau at 1616 COVID‐19 related deaths by 15 May 2020. New Jersey, by contrast, with a population of approximately 9 000 000 began social distancing measures on 18 March 2020 yet is projected to plateau at 3915 COVID‐19‐related deaths by 1 May 2020. Factors other than social distancing may in part explain this discrepancy.

These and other COVID‐19 epidemic models appear to assume a universal basic reproductive number (*R*_0_) for virus spread, as well as a uniform degree of pathogenicity. A recent report^2^ suggests the *R*_0_ of severe acute respiratory syndrome coronavirus 2 (SARS‐CoV‐2) may be as high as 5.7, increased from the original estimated *R*_0_ of 2.28.^3^ These reports also appear to assume uniform pathogenicity and transmission of the virus through time and do not account for mutation of the virus either towards or away from a more virulent strain.

As is typical of coronavirus, SARS‐CoV‐2 has rapidly acquired mutations allowing for tracking its ancestry and spread.^4^, ^5^ For example, an S type of the virus accounted for only 3.7% of viral isolates in Wuhan compared to 96.3% of the L type yet isolates outside of Wuhan were 61.3% L type and 38.4% S type.^4^ Examining clades of SARS‐CoV‐2 in 2310 geographically distributed viral DNA sequences using Nexstrain^5^ (Figure 1) reveal that the B1 clade predominates on the West Coast of the United States, and the A2a clade, which apparently spread to New York through Europe and Italy if this limited viral sequence phylogeny analysis is correct, predominates on the East Coast.

**Figure 1:**
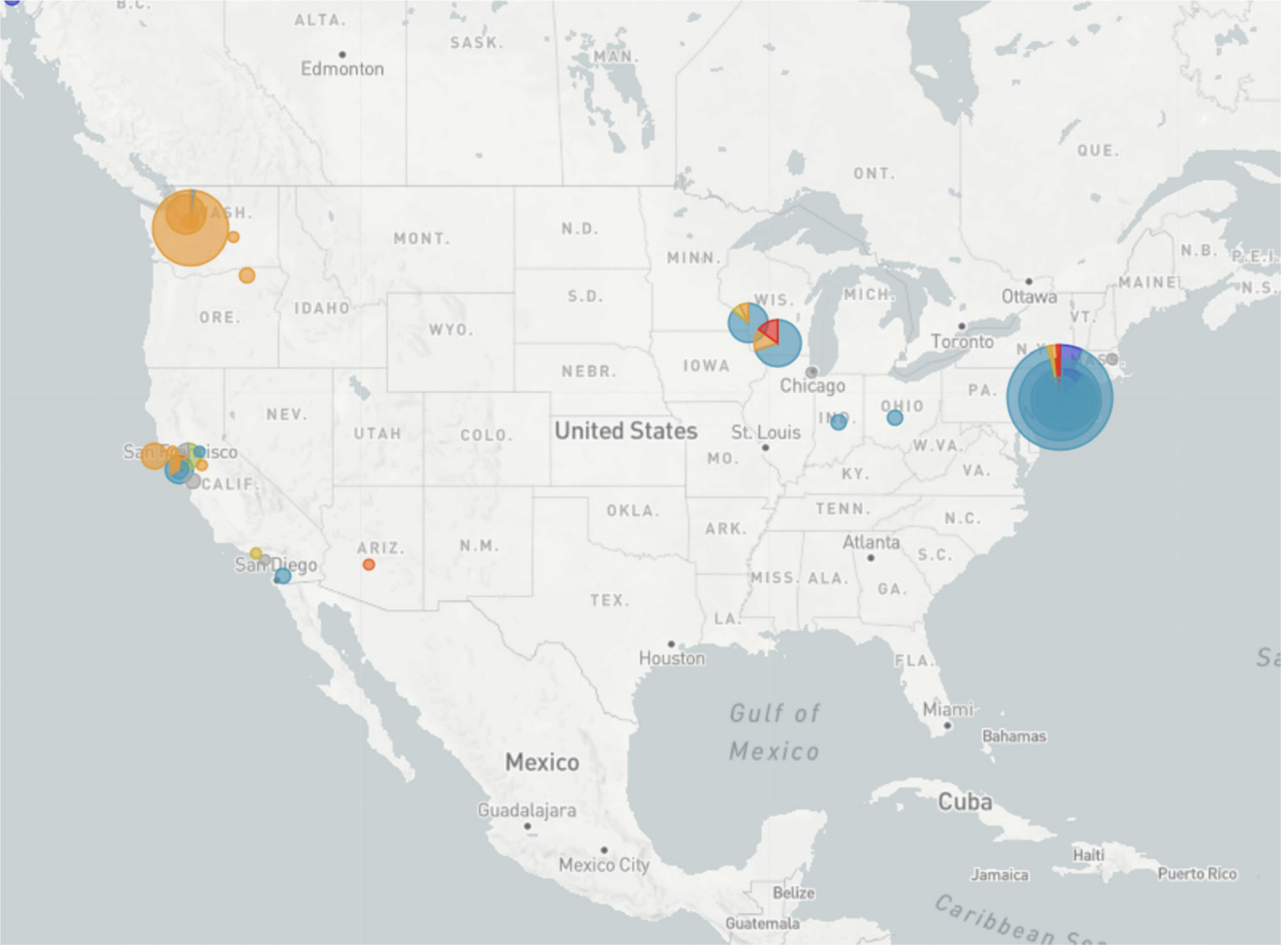
Dominant viral clades in United States clade B1 (orange) and clade A2a (blue) https://nextstrain.org/ncov/north-america?c=clade_membership&r=location. Accessed 15 April 2020

It has not escaped notice that these clades may vary in virulence. For example, analysis in Nexstrain in 2310 viral isolates of distribution of a nonsynonymous mutation in the viral spike protein at codon 614 (Figure 2) reveals that aspartic acid (D) at this residue is predominant on the West Coast and glycine (G) is predominant on the East Coast. SARS‐CoV‐2 genome phylogeny analysis reveals that this D614G mutation appeared to arise from an ancestral D residue (Figure 3). This mutation resides in a highly glycosylated region of the viral spike protein. A theory of viral pathogenesis^6^, as well as cryogeneiccryogenic electron microscopy^7^ and structure and function experiments^8^, suggest the possibility that mutations in this region could alter this heavily glycosylated viral spike and alter membrane fusion in tissues, resulting in more pathogenicity and more human to human spread. This residue is highly conserved in coronaviruses.^8^ There are many examples of mutation in this spike protein region resulting in changes in virulence,^9^, ^10^ and, in particular, a single serine to glycine change at residue 310 in a murine coronavirus spike protein resulted in increased virulence^11^ through decreased stability of the viral fusion machinery.

**Figure 2:**
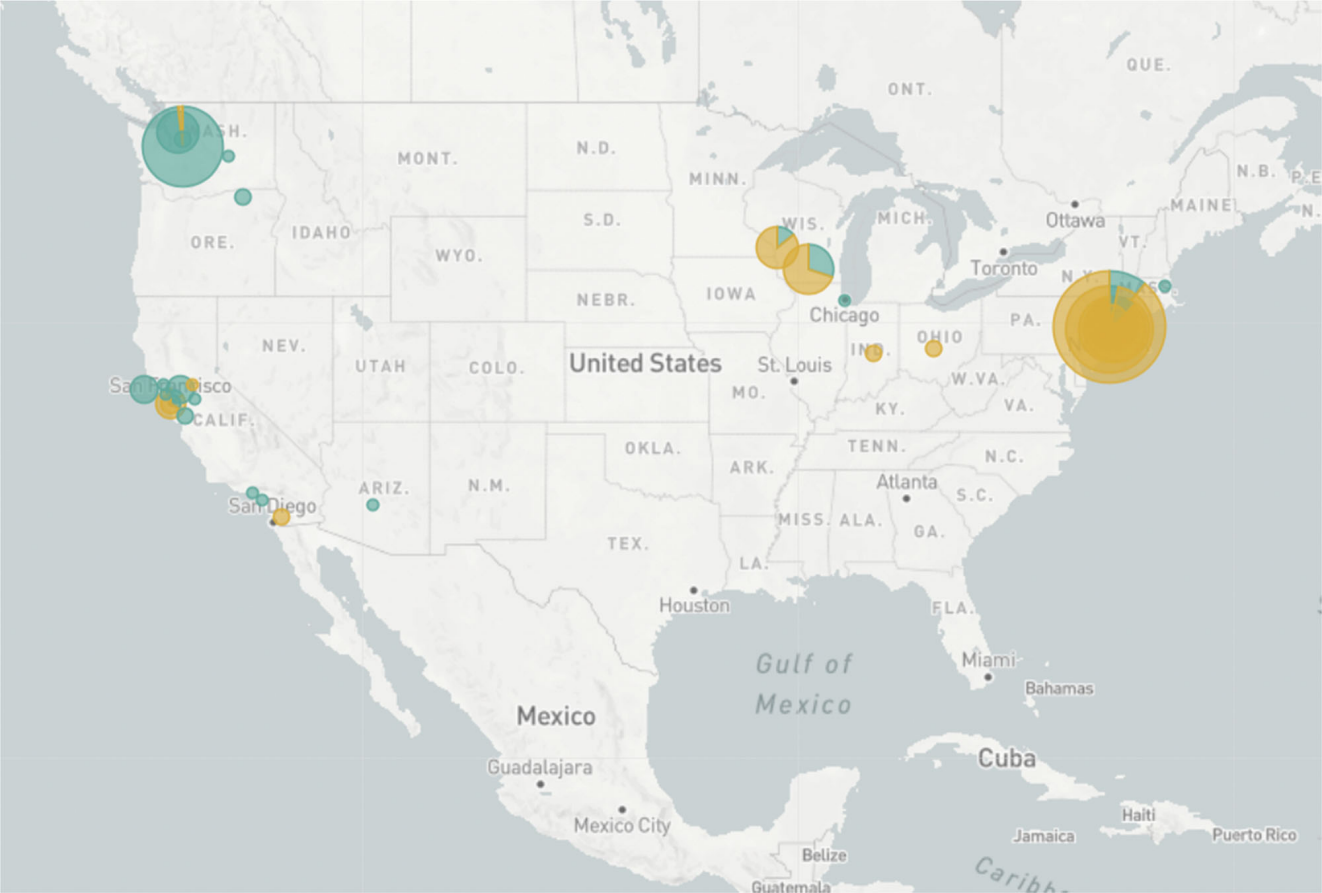
Distribution of codon 614 in the SARS‐CoV‐2 coronavirus aspartic acid (aqua), glycine (orange) https://nextstrain.org/ncov/north-america?c=gt-S_614&r=location. Accessed 15 April 2020. SARS‐CoV‐2, severe acute respiratory syndrome coronavirus 2

**Figure 3:**
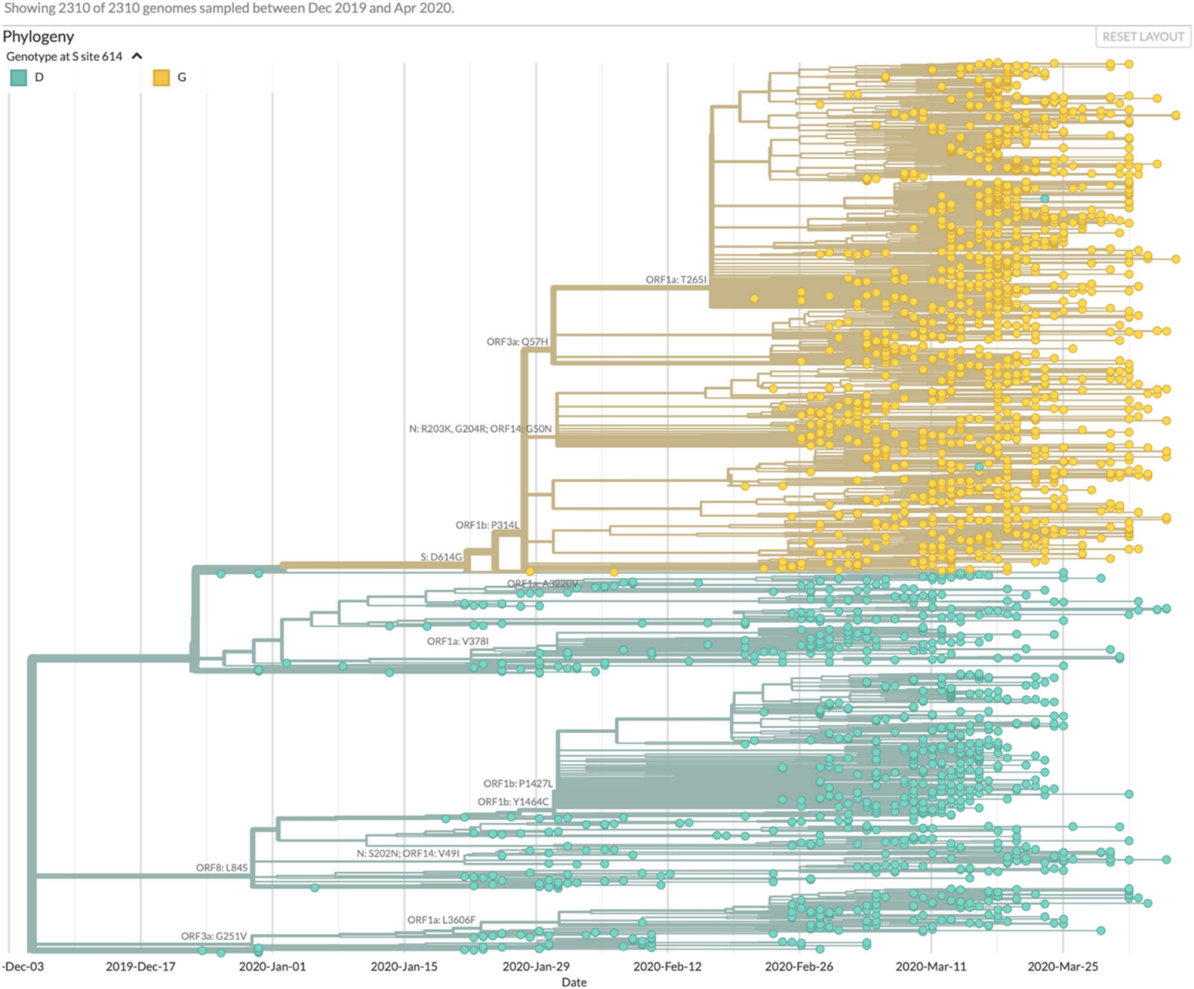
Inferred phylogeny of codon 614 spike protein mutation in SARS‐CoV‐2 https://nextstrain.org/ncov/north-america?branchLabel=aa&c=gt-S_614. Accessed 15 April 2020. SARS‐CoV‐2, severe acute respiratory syndrome coronavirus 2

This analysis has clear limitations. Virus phylogeny studies, for example, can have sampling bias as well as statistical bias in the inference model used.^12^ However, it should be relatively straightforward to test the virulence of various viral strains of SARS‐CoV‐2 in vitro, in particular those that have variability at residue 614 of the viral spike protein.

This suggests that competition between viral strains of varying virulence may be at play during the COVID‐19 pandemic. It is unclear whether current serologic or viral polymerase chain reaction‐based assays are able to detect this variability.^8^ If this hypothesis is correct, it may be important to develop assays based on local viral clades, to determine the distribution of the virus and its spectrum of effects. It may also be important for modelers to consider these possible variations in SARS‐CoV‐2 viral pathogenicity as models are developed for a gradual relaxation of social restrictions. In particular, differences and timing of the activation of innate or adaptive immunity to both viral strains as the pandemic progresses could be considered.

## CONFLICT OF INTERESTS

The authors declare that there are no conflict of interests.
